# Single-step nanoparticle antigen presentation system for tumor immunotherapy

**DOI:** 10.1186/2051-1426-3-S2-P319

**Published:** 2015-11-04

**Authors:** Frederick Kohlhapp, Erica Huelsmann, Jai Rudra, Arman Nabatiyan, Andrew Zloza

**Affiliations:** 1 Rutgers CINJ, New Brunswick, NJ, USA; 2 Rush University, Chicago, IL, USA; 3 UTMB, Galveston, TX, USA

## Background and aim

While only 10-30% of patients treated with currently approved single immunotherapies demonstrate long-term benefit, treatment efficacy in responders correlates with increased CD8+ T cell responses to neoantigens (tumor antigens hidden from the immune system). Thus, to improve presentation of tumor antigens and expose neoantigens we engineered a single-step nanoparticle antigen presentation system (SNAPS) that enables both membrane and cytosolic cancer proteins to be covalently coupled to an injectable nanoparticle emulsion.

## Methods

B16 melanoma (SNAPS-B16), primary melanocyte (SNAPS-MEL), 4T1 breast cancer (SNAPS-4T1) lysate or mouse IgG (SNAPS-C) were used for coating polystyrene nanoparticles via an EDAC linker. One day or 11 days post B16-F10 tumor cell challenge (100,000 cells, intradermal, right flank), mice were treated (at the same site as the tumor cell injection) with 200 μL of 1% w/v cell lysate coated nanoparticles. Tumors were measured using calipers every 2-3 days. Flow cytometry staining was performed for antigen presenting cells (APCs) and CD8+ T cells after dissection and mechanical dissociation of the tumor. Statistical analyses were performed using the student t test and Prism v4.0 software (GraphPad). Differences with a P < 0.05 were considered statistically significant.

## Results

We observed that SNAPS-B16 delivered at one day post tumor challenge significantly reduced tumor growth (P < 0.01) compared to SNAPS-C (which had no effect). SNAPS delivered at day 11 (when tumors were approximately 5 mm × 5 mm) significantly halted tumor growth (< 2-fold increase over the course of 10 days) while B16 treated with SNAPS-C increased ~16-fold (P < 0.01). Neither SNAPS-4T1 nor SNAPS-MEL had any effect on the growth of B16 *in vivo*, demonstrating the induction of an antigen-specific anti-tumor response with SNAPS-B16. Additionally, SNAPS-B16 significantly increased (P < 0.01) the proportion of APCs within the tumor microenvironment compared to the SNAPS-C, as evidenced by the increased percentage of MHC-II+ cells).

## Conclusions

Our results demonstrate that SNAPS coupling of whole tumor lysate from cancer cells to nanoparticles delivers a robust anti-tumor immune response resulting in enhanced tumor regression. These findings demonstrate that a cancer-derived protein unique to B16 melanoma (rather than to tumors in general or to normal melanocytes) coating the SNAPS confers tumor specificity. Although preliminary, our reported innovation of using nanoparticles coated through EDAC with tumor proteins in this specific manner to elicit immune responses against tumor cells *in vivo* may sidestep many of the complexities and per patient costs associated with current cell-based immunotherapies and tumor antigen sequencing.

